# Exploring the link between poor oral hygiene and mesh infection after hernia repair: a systematic review and proposed best practices

**DOI:** 10.1007/s10029-023-02795-y

**Published:** 2023-05-19

**Authors:** B. East, M. Podda, M. Beznosková-Seydlová, A. C. de Beaux

**Affiliations:** 1 3rd Department of Surgery of 1st Faculty of Medicine at Charles University, Motol University Hospital, Prague, Czech Republic; 2 Department of Surgical Science, Emergency Surgery Unit, University of Cagliari, Cagliari, Italy; 3 Private dentist, Prague, Czech Republic; 4 Spire Murrayfield Hospital, Edinburgh and The University of Edinburgh, Edinburgh, UK

**Keywords:** Hernia, Mesh, Infection, Oral/dental health

## Abstract

**Background:**

There is a reasonable body of evidence around oral/dental health and implant infection in orthopaedic and cardiovascular surgery. Another large area of surgical practice associated with a permanent implant is mesh hernia repair. This study aimed to review the evidence around oral/dental health and mesh infection.

**Methods:**

The research protocol was registered in PROSPERO (CRD42022334530). A systematic review of the literature was undertaken according to the PRISMA 2020 statement. The initial search identified 582 publications. A further four papers were identified from references. After a review by title and abstract, 40 papers were read in full text. Fourteen publications were included in the final review, and a total of 47,486 patients were included.

**Results:**

There is no published evidence investigating the state of oral hygiene/health and the risk of mesh infection or other infections in hernia surgery. Improvement in oral hygiene/health can reduce surgical site infection and implant infection in colorectal, gastric, liver, orthopaedic and cardiovascular surgery. Poor oral hygiene/health is associated with a large increase in oral bacteria and bacteraemia in everyday activities such as when chewing or brushing teeth. Antibiotic prophylaxis does not appear to be necessary before invasive dental care in patients with an implant.

**Conclusion:**

Good oral hygiene and oral health is a strong public health message. The effect of poor oral hygiene on mesh infection and other complications of mesh hernia repair is unknown. While research is clearly needed in this area, extrapolating from evidence in other areas of surgery where implants are used, good oral hygiene/health should be encouraged amongst hernia patients both prior to and after their surgery.

**Supplementary Information:**

The online version contains supplementary material available at 10.1007/s10029-023-02795-y.

## Introduction

The role of perioperative antibiotics in operations where a foreign body is implanted has become common good practice. Similarly, many specialties utilising implants, such as orthopaedic and cardiac surgery, screen patients for possible dental focal infections, including gum disease. There is good evidence that even the cleaning of teeth in the presence of poor oral health is associated with significant episodes of transient bacteraemia [1–4]. While pre-optimisation of patients prior to more complex abdominal wall repair is becoming more popular [5], we are not aware that poor oral hygiene/health has been taken into consideration as part of prehabilitation. In addition to infection at the time of mesh implant, there is also a question about the possibility of mesh infection months or years after the mesh implant as a consequence of oral bacteria related to poor oral hygiene/health. Oral hygiene is a term that encompasses a number of facets. This includes the regular cleaning (brushing/flossing) of teeth to minimise plaque accumulation, the care of gums and tongue, as well as regular dental review to treat dental caries and mouth infections. Oral health is a term related to the presence or absence of tooth, gum or tongue disease.

Some of the authors of this paper (BE, AdeB) were involved in a world-wide based survey of the attitudes of patients to aspects of hernia surgery [6]. A number of patients commented in the free text about mesh problems, mesh infection, and ending up losing some of their teeth. Indeed, a number were in a legal dispute with mesh manufacturers and surgeons due to loss of teeth following hernia repair with mesh. The same people also suffered a mesh infection. There is no obvious causative reason why a mesh may lead to the loss of one or more teeth. But there is the possibility that poor oral hygiene and poor oral health, including dental focal infection, could cause perioperative or postoperative bacteraemia leading to subsequent mesh infection. The tooth loss was a consequence of ongoing tooth pathology and was not caused by the mesh hernia repair at all.

The aim of this study was to undertake a systematic review of oral hygiene/health and mesh hernia surgery. However, an initial scoping search looking for publications on mesh infection and oral/dental health/pathology identified no references. Thus, the search was widened to oral hygiene/health and distant hematogenous infections after surgery, to inform on the potential issue of implant infection as a result of poor oral hygiene/health.

## Materials and methods

The systematic review (SR) was registered in the PROSPERO register (CRD42022334530).

MEDLINE (via PubMed), EMBASE, and the Cochrane Central Register of Controlled Trials databases were searched. The search strategy combined medical subject headings (MeSH) and keywords, using the terms of “Dental”, “Oral”, “Health”, “Infection”, “Cardiac”, “Orthopaedic”, “Abdominal”, “Hernia”, “Mesh” and “Surgery” combined with the Boolean operators “AND” and “OR” (Appendix 1). To begin with, we planned to focus the SR on patients undergoing hernia surgery with mesh only, but a scoping review revealed no results. We have, therefore, decided to widen our inclusion criteria.

The following PICO (Patient, Intervention, Control, Outcome) question was adopted:

P (Patient): Patients undergoing any type of operation that involves a permanent synthetic implant.

I (Intervention): Any of the following interventions were inclusion criteria into the SR—Patients being given instructions to change practice in teeth brushing, check by a dentist prior to or after the operation, perioperative mouth care, dental hygienist treatment before the operation or during the follow-up period, screening for potential foci of infection in the oral cavity pre/post operatively.

C (Comparison): No such intervention or investigation, no change in practice, no focus on dental/oral health/hygiene.

O (Outcome): Occurrence of implant infection or distant SSI that is not due to direct spreading from the oral cavity.

Each phase of the systematic review (literature search, data extraction, and risk of bias assessment) was performed by two authors (BE and MP). Discrepancies were resolved with discussion with a third reviewer (AdB) and a dentist (MBS). The GRADE approach^7^ was followed. Two authors independently (MP and BE) evaluated the evidence for imprecision, inconsistency, indirectness, and publication bias. The quality of evidence was classified as very low, low, moderate, or high. The risk of bias in the included studies was independently assessed by two authors (BE and AdB) using the ROBINS-I (Risk Of Bias In Non-randomised Studies—of Interventions) tool [8].

Statistical analysis was not performed on the retrieved data. Randomised controlled trials (RCT), retrospective, case–control, or prospective observational studies (OS), case series (CS), and systematic reviews (SR) exploring the potential association between oral hygiene and hematogenous distant infection were deemed suitable for inclusion in the review. We have limited our search to only adult human subjects.

No restrictions were placed on publication status or language. Full-text articles in languages other than English with a title/abstract indicating fulfilment of the eligibility criteria were translated electronically. Literature was searched from inception to April 2022. The studies identified by the search strategy were subsequently selected based on title, abstract, and full-text review by all four independent reviewers in the Rayyan web app for systematic reviews ( https://www.rayyan.ai/).

## Results

Upon analysis of the retrieved literature, we discovered that the studies report on the same outcome but with two different timings of the intervention: some studies focus on oral/dental intervention before the index operation, while others examine dental/oral interventions after implant surgery. As a result, we have decided to split the results section into two chapters to address each intervention timing separately.

**Fig. 1:**
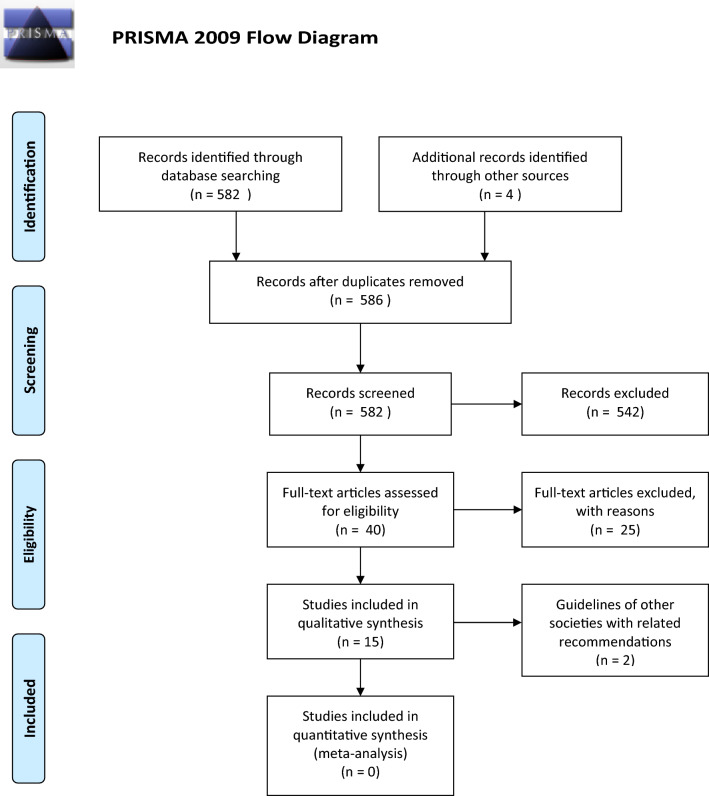
Prisma 2009 flow diagram

The literature search identified 582 papers. Four more references were identified by manual search through individual article references. Figure 1 displays the 586 papers included in the Prisma flowchart. Forty papers were retrieved in full text, and 14 were kept for final analysis. A table with rejected papers and reasons for rejection is attached as Supplementary Table 1. Two of the included publications were recommendations from other guidelines, the 14 remaining studies report on 47,486 patients in total.

No papers reported on the topic of oral hygiene/health and hernia mesh infection or surgical site infection (SSI) in hernia surgery. However, papers investigating the link between oral hygiene/health prior to surgical implants in other areas of surgery were identified. And papers around the use of antibiotics prior to dental manipulation in patients with artificial joints, those at risk of infective endocarditis (IE) and with artificial heart valves, and hematogenous infections were identified.

The risk of bias was low to moderate in the majority of the selected studies (Supplementary Table 2), but mainly due to the type of the studies, the quality of evidence was in most cases low or even very low (Supplementary Table 3, 4, 5).

### Does preoperative improvement of oral hygiene/health before (implant) surgery improve infective outcomes?

One recent SR (Supplementary material—Table 3) in orthopaedic joint implants and another in cardiac valve replacement surgery (CVS) reported a lack of strong evidence to suggest that preoperative dental treatment improves patients outcomes [9, 10]. Despite being SR both were rated as low quality, especially due to the included studies. However, three recent prospective cohort studies (Supplementary material—Table 4) demonstrated the importance of oral hygiene prior to cardiac procedures and its impact on post-procedure antibiotic use, postoperative fever, and SSI [11–13]. A study with 240 participants reported that the identification and treatment of dental foci prior to cardiac surgery was able to significantly reduce the overall infection rate (4/55 vs. 3/185; 7.27% vs. 1.62%; *p* = 0.062) but also slightly reduce the leukocyte count (10.98 vs. 10.13 × 103/μL, *p* = 0.061) in the sub-cohort with no infection complication. Incomplete oral sanitation with odds ratio (OR) of 6.1 (*p* = 0.042) was together with diabetes mellitus (OR 5.38, *p* = 0.059), the most important risk factor for infectious complications in this group [11].

A second study looked at the effect of a recommendation on how to brush teeth and perform mouth washing in a cohort of 466 patients undergoing cardiac surgery compared to 506 historical controls. 405 were able to adhere to the recommendations, and there were no demographic differences between them and the control group. Significantly, fewer patients had been prescribed antibiotics on the fifth postoperative day in the intervention group (*p* < 0.015, RR = 0.65 (95% CI 0.48 to 0.96), number needed to treat (NNT) 22.0 patients, relative risk reduction (RRR) 0.52, absolute risk reduction (ARR) 0.042) and the results were even more significant amongst patients who had adhered to the procedure (RR = 0.49 (95% CI 0.31 to 0.77), NNT = 15.9, RRR = 1.01, ARR = 0.063) compared with patients in the control group. Thirty-four % fewer people needed antibiotics only after being told how to brush their teeth, and this number rose to 50% in the group able to adhere to the recommendation [12].

The third of the mentioned studies looked at a small group of 64 patients undergoing cardiac surgery versus 38 historical controls. Patients in the study have undergone preoperative periodontal treatment compared to the historical controls. The number of days people from the intervention group suffered from a temperature over 37.5 °C after their operation was significantly lower than in the control group (*p* = 0.01) [13].

A further study demonstrated the link between the severity of periodontal disease and surgical outcomes of patients undergoing total gastrectomy [14]. Twenty-six out of 52 patients had the same bacteria in their gastric mucosa as in their oral cavity despite prophylactic antibiotic administration. The other 50% of patients had only mild periodontal disease.

Four recent retrospective cohort series [15–18] in colorectal, gastric and liver surgery all demonstrated reductions in postoperative infective events, with perioperative oral hygiene interventions (Supplementary material—Table 3).

Two studies in colorectal surgery reported on one with 698 and the other one with 1926 patients and showed that perioperative oral management is able to reduce the incidence of SSI and shorten the hospital stay significantly. The incidence of surgical site infection was significantly lower in the oral care group than in the control group (8.4% vs 15.7%, *P* < 0.001). Multivariate logistic regression analysis revealed four independent risk factors for surgical site infection: low albumin level, rectal cancer, blood loss, and lack of perioperative oral care. Lack of perioperative oral care had an OR of 2.100 (95% confidence interval 1.510–2.930, *P* < 0.001) [16, 17].

A single institution's historical experience with dentists assessing the oral environment for periodontal disease, hygiene status, dry mouth, fur on tongue, and tooth stumps, scaling and tooth brushing instructions included 341 patients. Logistic regression analysis identified periodontal disease as an independent risk factor for postoperative infectious complication with OR of 2.091 (*p* = 0.037, 95% confidence interval 1.045–4.183) [15]. Similarly, an experience from 334 patients undergoing hepatic resection where independent risk factors for SSI were infection of ascites (OR = 13.72), lack of preoperative oral management intervention (OR = 10.17), and severe liver fibrosis (OR = 2.76) [18].

### Does dental treatment after implant surgery influence late implant infection?

It is believed that dental surgery causes significant bacteraemia and, therefore, dental manipulation after implant surgery might cause implant infection and that prophylactic antibiotics might mitigate this risk. Again, the most recent SRs and a guideline in the orthopaedic and cardiovascular surgical fields would suggest that it is not necessary to give antibiotics at the time of dental surgery [20–22]. These are well supported by the evidence provided by three retrospective population studies from three different continents [23–25].

A Taiwanese population-based analysis screened 57 066 hip and knee arthroplasty recipients that had dental workup. A propensity score analysis (of 6 513 matched pairs) compared those with and without prophylactic antibiotics for their dental care. Infection occurred in 328 (0.57%) in the dental sub-cohort and 348 (0.61%) patients in the non-dental sub-cohort with no between-cohort difference in the 1-year cumulative incidence (0.6% in both, *P* = 0.3). Multivariate-adjusted Cox regression revealed no association between dental procedures and joint infection. Maybe more interestingly, infection occurred in 13 patients (0.2%) in those who received antibiotics and 12 (0.18%) of those who did not (*P* = 0.8). Multivariate-adjusted analyses confirmed that there was no association between the incidence of prosthetic joint infection and prophylactic antibiotics [23].

Medicare-Based Survey (MCBS) using data for the years 1997 to 2006 identified patients undergoing total joint arthroplasty and those who have suffered from prosthetic joint infection. A time-to-event analysis (*N* = 1 000) was utilised to explore association between dental procedures and subsequent joint infection. A nested case–control study included case participants who had had prosthetic joint infection (*n* = 42) and matched control participants who had had total arthroplasty but had no infection (*n* = 126). People with no infection were more likely to have undergone invasive dental procedure though this trend was not statistically significant in either the time-to-event analysis (HR = 0.78; 95% CI 0.18–3.39) or the case–control analysis (OR = 0.56; 95% CI 0.18–1.74). Only 4 of 42 case participants had undergone an invasive dental procedure in the 90 days before the infection occurred. All dental procedures yielded similar results [24]. A third study also reported hospital admissions and people with prosthetic joint infections and their dental records. 9427 cases were identified—2 385 had prosthetic hip, 3 168 knee, 3 615 unknown type and 259 other joint replacements. There was no significant temporal association between invasive dental procedures and subsequent late prosthetic joint infection rate [25].

## Discussion

While dental surgery does cause significant bacteraemia, which can be partially mitigated against by prophylactic antibiotics, there is significant bacteraemia when teeth cleaning and chewing every day, events that are not covered with antibiotics [1–4]. Yet, the Society of Vascular Surgery recommends that antibiotics are given following a vascular graft prior to dental surgery [19]. A number of French Societies produced a guideline [26] on infective endocarditis prevention in those at risk such as valvular heart disease. About one-fifth of infective endocarditis is believed to be due to oral bacteria, not as a result of dental manipulation but daily activities leading to low-dose but long-lasting bacteriemia events. Improved oral hygiene/health may reduce the incidence of infective endocarditis in this at-risk population.

Guidelines for both orthopaedic and cardiovascular implant surgery recommend assessing the state of oral health, eliminating all dental focal infections and improving oral hygiene where necessary prior to implant surgery. Indeed, improving oral health is now standard practice in many countries prior to orthopaedic prosthetic joint replacements and cardiovascular implants. The Society of Vascular Surgery for example, recommends in their guidelines [19] that potential sources of dental sepsis are treated at least 2 weeks prior to vascular surgery.

This study has demonstrated that there is no published research on oral hygiene/health in patients undergoing hernia repair with mesh implants. Thus, the role of improving oral hygiene/health prior to mesh hernia surgery, or the long-term effect of poor oral health or dental focal infection relating to late mesh infection is unknown. However, it is evident that episodes of bacteraemia take place on chewing, teeth cleaning when bad oral health is present and also during and after dental surgery [1–4]. The number and degree of bacteraemia events appears to be increased in the presence of poor oral hygiene/health. And the prevalence of poor oral health is high in general throughout the world [27, 28]. Šutej et al. have pointed out a high percentage of patients with infective endocarditis whose infection originated in the oral cavity [29] and Strom et al. reported that daily flossing can reduce the risk of IE significantly [30].

While other surgical disciplines (cardiovascular, orthopaedics) are diligent in eliminating any potential infection focus prior to any use of permanent implants, including poor oral health, mesh hernia surgery has not followed this standard of care. Both early and late mesh infections occur, and are often associated with poor outcomes for both the healthcare service AND the patient. Looking at the types of bacteria cultured from these mesh infections, the patients' skin microbiome is often blamed as the source or reservoir for the infecting bacteria. And colonisation of the mesh at the time of surgery, with a latent period before overt infection considered the mechanism in late mesh infection. The fact that microorganisms typical for skin also live in the periodontal plaque and circulate in our blood every time we chew or brush our teeth may change this dogma [31]. However, more research is required to identify a possible link between poor oral health and hernia mesh infection. And if there is a link, to investigate the cost-effectiveness of preoperative improvement in oral hygiene/health prior to mesh hernia surgery. Based on the literature available to date in other surgical fields, we would like to suggest that patients undergoing mesh hernia surgery should undergo a dental check-up and treatment of any tooth or gum disease prior to their hernia surgery. Furthermore, advice on how to improve oral hygiene should be part of the pre-assessment and patient work-up process. Periodontal disease is common in the population, especially with increasing age. The majority of hernia surgery is undertaken as an elective procedure, with time for proper oral health assessment and treatment where necessary.

There is evidence that poor oral hygiene/health is linked to long-term adverse outcomes in other fields of surgery and medicine. There has been a lot of research interest around the use of prophylactic antibiotics prior to dental surgery following surgical implants. Little benefit if any has been demonstrated for this practice. And the cost and potential harms with emerging antibiotic resistance, and antibiotic drug reactions are a strong argument against such practice. Therefore, it is not recommended to use antibiotics prophylactically before dental surgery in patients with medical implants. Yet concerns around medico-legal claims for example, infected joint replacements after dental treatment continue to encourage the use of antibiotics in this scenario [20]. Furthermore, compliance with both antibiotic type, dosing, and indication guidelines remains poor [32–34]. It is likely that dental interventions as a cause of bacteraemia are rare compared to those associated with daily activities including chewing and the brushing of teeth. In fact, as shown by Skaar et al. in their population study people who undergo dental procedures are less likely to suffer periprosthetic joint infections compared to controls [24]. Activities like brushing or chewing are also associated with significant episodes of bacteraemia, which are not covered with antibiotic prophylaxis! Therefore, maintaining good oral hygiene and health through daily brushing and flossing is essential, as it can reduce the risk of infection and improve overall health.

Our study has a number of limitations. There is a publication bias in reporting single centre experiences and retrospective cohort studies, as many cases of distant implant infection are likely not reported, and the denominator in these studies is often unclear also. It is likely that the real magnitude of the problem is much higher than the literature suggests. Only a fraction, although a very significant one, of all cases of hematogenous infection of various implants are identified as “surely” of odontogenic source. But as shown by Zawadski et al. [31], the oral cavity is a source of many other bacterial strains than those found uniquely in the human mouth. Periodontal plaque is common. It is associated with frailty, as well as lowered health literacy, and these may be confounding variables when investigating oral hygiene/health on surgical outcomes [35].

While no study reporting on a link between oral hygiene/health and hernia mesh infection was identified, it is possible that any episode of bacteraemia can lead to the growth of a biofilm on such a mesh implant, leading to clinically apparent mesh infection. The patient may well link such infection of their mesh to their dental issues and come to a conclusion that the mesh is responsible for their tooth and gum disease. It is perhaps more probable than the sequence of events is the other way around—their tooth and gum disease (which could be occult at the time of their hernia surgery if regular dental checks are not undertaken) is the cause of their hernia mesh infection.

It should be stressed that improving oral hygiene/health has few if any negative or harmful effects on these individuals. It is noted that dental health care is not free in most countries, and there is a small cost to daily good oral hygiene. But our proposed change of practice, to include advice on oral hygiene, and check the state of oral health when considering a surgical mesh implant in hernia is unlikely to have any serious negative consequences for the patient. Indeed, it is important public health advice that may benefit the patient in other ways by reducing disease associated with poor oral health.

Further research is, therefore, very likely to have an important impact on our confidence in the estimate of effect.

## Suggestion for future research

Mesh hernia surgery is the commonest general surgical operation. Further research into the possible association between oral health and both early and late mesh infection, as well as SSI should be a priority. There are hernia registries that could add a function of collecting oral/dental health data and intervention status. This would be the easiest way to collect a big amount of data in a relatively short time. Also, some countries that have national based registries of all interventions and treatments could perform an analysis similar to some of those mentioned in this manuscript. The EHS registry will add this functionality in the near future. A prospective trial looking into dental status and interventions and corresponding incidence of mesh infection would be another step forward in patient’s pre-optimisation knowledgebase.

### Electronic supplementary material

Below is the link to the electronic supplementary material.

Supplementary file1 (DOCX 17 KB)

Supplementary file2 (DOC 43 KB)

Supplementary file3 (DOCX 19 KB)

Supplementary file4 (DOCX 16 KB)

Supplementary file5 (DOCX 18 KB)

## Appendix_1 Search strategies

1. (((((((oral hygiene) OR (dental hygiene)) AND (mesh infection)) OR (prosthesis infection)) OR (hematogenous infection)) AND (cardiovascular surgery)) OR (orthopedic surgery)) OR (hernia surgery) (((("oral hygiene"[Title] OR "dental hygiene"[Title]) AND "mesh infection"[Title]) OR "prosthesis infection"[Title] OR "hematogenous infection"[Title]) AND "cardiovascular surgery"[Title]) OR "orthopedic surgery"[Title] OR "hernia surgery"[Title]
2. (((((((oral hygiene[MeSH Terms]) OR (dental hygiene[MeSH Terms])) AND (mesh infection[MeSH Terms])) OR (prosthesis infection[MeSH Terms])) OR (hematogenous infection[MeSH Terms])) AND (cardiovascular surgery[MeSH Terms])) OR (orthopedic surgery[MeSH Terms])) OR (hernia surgery[MeSH Terms]) Filters: from 2000 - 2023 Sort by: Most Recent ((((("oral hygiene"[MeSH Terms] OR "oral hygiene"[MeSH Terms]) AND (("medical subject headings"[MeSH Terms] OR ("medical"[All Fields] AND "subject"[All Fields] AND "headings"[All Fields]) OR "medical subject headings"[All Fields] OR "mesh"[All Fields]) AND "infections"[MeSH Terms])) OR (("prostheses and implants"[MeSH Terms] OR ("prostheses"[All Fields] AND "implants"[All Fields]) OR "prostheses and implants"[All Fields] OR "prosthesis"[All Fields]) AND "infections"[MeSH Terms]) OR (("haematogeneous"[All Fields] OR "haematogenic"[All Fields] OR "haematogenous"[All Fields] OR "haematogenously"[All Fields] OR "hematogeneous"[All Fields] OR "hematogeneously"[All Fields] OR "hematogenic"[All Fields] OR "hematogenous"[All Fields] OR "hematogenously"[All Fields]) AND "infections"[MeSH Terms])) AND "cardiovascular surgical procedures"[MeSH Terms]) OR "orthopedics"[MeSH Terms] OR (("hernia"[MeSH Terms] OR "hernia"[All Fields] OR "hernias"[All Fields] OR "hernia s"[All Fields] OR "herniae"[All Fields]) AND ("surgical procedures, operative"[MeSH Terms] OR "general surgery"[MeSH Terms]))) AND (2000:2023[pdat])

## Translations

1. oral hygiene[MeSH Terms]: "oral hygiene"[MeSH Terms] dental hygiene[MeSH Terms]: "oral hygiene"[MeSH Terms] mesh: "medical subject headings"[MeSH Terms] OR ("medical"[All Fields] AND "subject"[All Fields] AND "headings"[All Fields]) OR "medical subject headings"[All Fields] OR "mesh"[All Fields] infection[MeSH Terms]: "infections"[MeSH Terms] prosthesis: "prostheses and implants"[MeSH Terms] OR ("prostheses"[All Fields] AND "implants"[All Fields]) OR "prostheses and implants"[All Fields] OR "prosthesis"[All Fields] OR "prosthesis's"[All Fields] infection[MeSH Terms]: "infections"[MeSH Terms] hematogenous: "haematogeneous"[All Fields] OR "haematogenic"[All Fields] OR "haematogenous"[All Fields] OR "haematogenously"[All Fields] OR "hematogeneous"[All Fields] OR "hematogeneously"[All Fields] OR "hematogenic"[All Fields] OR "hematogenous"[All Fields] OR "hematogenously"[All Fields] infection[MeSH Terms]: "infections"[MeSH Terms] cardiovascular surgery[MeSH Terms]: "cardiovascular surgical procedures"[MeSH Terms] orthopedic surgery[MeSH Terms]: "orthopedics"[MeSH Terms] hernia: "hernia"[MeSH Terms] OR "hernia"[All Fields] OR "hernias"[All Fields] OR "hernia's"[All Fields] OR "herniae"[All Fields] surgery[MeSH Terms]: "surgical procedures, operative"[MeSH Terms] OR "general surgery"[MeSH Terms]

## References

[1] Guntheroth WG (1984). How important are dental procedures as a cause of infective endocarditis?. Am J Cardiol.

[2] Kinane DF, Riggio MP, Walker KF, MacKenzie D, Shearer B (2005). Bacteraemia following periodontal procedures. J Clin Periodontol.

[3] Lockhart PB, Brennan MT, Sasser HC, Fox PC, Paster BJ, Bahrani-Mougeot FK (2008). Bacteremia associated with toothbrushing and dental extraction. Circulation.

[4] Forner L, Larsen T, Kilian M, Holmstrup P (2006). Incidence of bacteremia after chewing, tooth brushing and scaling in individuals with periodontal inflammation. J Clin Periodontol.

[5] Jensen KK, East B, Jisova B, Cano ML, Cavallaro G, Jørgensen LN, Rodrigues V, Stabilini C, Wouters D, Berrevoet F. The European Hernia Society Prehabilitation Project: a systematic review of patient prehabilitation prior to ventral hernia surgery. Hernia. 2022 Feb 25. doi: 0.1007/s10029–022–02573–2. Epub ahead of print. PMID: 35212807. 10.1007/s10029-022-02573-2 35212807

[6] East B, Hill S, Dames N, Blackwell S, Laidlaw L, Gök H, Stabilini C, de Beaux A (2021). Patient views around their hernia surgery: a worldwide online survey promoted through social media. Frontiers Surg.

[7] GRADEpro GDT: GRADEpro Guideline Development Tool [Software]. McMaster University, 2020 (developed by Evidence Prime, Inc.) https://gradepro.org/cite/gradepro.org

[8] Sterne JA, Hernán MA, Reeves BC, Savović J, Berkman ND, Viswanathan M, Henry D, Altman DG, Ansari MT, Boutron I, Carpenter JR, Chan AW, Churchill R, Deeks JJ, Hróbjartsson A, Kirkham J, Jüni P, Loke YK, Pigott TD, Ramsay CR, Higgins JP (2016). ROBINS-I: a tool for assessing risk of bias in non-randomised studies of interventions. BMJ (Clinical research ed).

[9] Barrere S, Reina N, Peters OA, Rapp L, Vergnes JN, Maret D (2019). Dental assessment prior to orthopedic surgery: a systematic review. Orthopaedics Traumatol, Surgery Res: OTSR.

[10] Lockhart PB, DeLong HR, Lipman RD, Abt E, Baddour LM, Colvin M, Miller CS, Sollecito T, O'Brien K, Estrich CG, Araujo MWB, Carrasco-Labra A (2019). Effect of dental treatment before cardiac valve surgery: systematic review and meta-analysis. J Am Dental Associat.

[11] Konstanty-Kalandyk J, Kalandyk-Konstanty A, Kapelak B, Zarzecka J, Drwila R, Kieltyka A, Piątek J, Bartuś K, Sadowski J (2016). Incomplete oral sanation as a risk factor for elevated leucocytosis and postoperative infection. Kardiol Pol.

[12] Pedersen PU, Tracey A, Sindby JE, Bjerrum M (2019). Preoperative oral hygiene recommendation before open-heart surgery: patients’ adherence and reduction of infections: a quality improvement study. BMJ Open Quality.

[13] Suzuki H, Matsuo K, Okamoto M, Nakata H, Sakamoto H, Fujita M (2019). Preoperative periodontal treatment and its effects on postoperative infection in cardiac valve surgery. Clinical Exp Dental Res.

[14] Nishikawa M, Honda M, Kimura R, Kobayashi A, Yamaguchi Y, Hori S, Kobayashi H, Waragai M, Kawamura H, Nakayama Y, Todate Y, Takano Y, Yamaguchi H, Hamada K, Iketani S, Seto I, Izumi Y, Seto K (2020). The bacterial association with oral cavity and intra-abdominal abscess after gastrectomy. PLoS ONE.

[15] Nishikawa M, Honda M, Kimura R, Kobayashi A, Yamaguchi Y, Kobayashi H, Kawamura H, Nakayama Y, Todate Y, Takano Y, Yamaguchi H, Hamada K, Iketani S, Seto I, Seto K (2019). Clinical impact of periodontal disease on postoperative complications in gastrointestinal cancer patients. Int J Clin Oncol.

[16] Nobuhara H, Yanamoto S, Funahara M, Matsugu Y, Hayashida S, Soutome S, Kawakita A, Ikeda S, Itamoto T, Umeda M (2018). Effect of perioperative oral management on the prevention of surgical site infection after colorectal cancer surgery: a multicenter retrospective analysis of 698 patients via analysis of covariance using propensity score. Medicine.

[17] Nobuhara H, Matsugu Y, Soutome S, Hayashida S, Hasegawa T, Akashi M, Yamada SI, Kurita H, Nakahara H, Nakahara M, Ueda N, Kirita T, Nakamura T, Shibuya Y, Mori K, Yamaguchi T (2022). Perioperative oral care can prevent surgical site infection after colorectal cancer surgery: a multicenter, retrospective study of 1,926 cases analyzed by propensity score matching. Surgery.

[18] Hasegawa T, Takeda D, Tanaka M, Amano R, Saito I, Kakei Y, Kimoto A, Fukumoto T, Akashi M (2021). Effects of preoperative dental examination and oral hygiene instruction on surgical site infection after hepatectomy: a retrospective study. Supportive Cancer : Off J Multinational Assoc Support Care Cancer.

[19] Chaikof EL, Dalman RL, Eskandari MK, Jackson BM, Lee WA, Mansour MA, Mastracci TM, Mell M, Murad MH, Nguyen LL, Oderich GS, Patel MS, Schermerhorn ML, Starnes BW (2018). The Society for Vascular Surgery practice guidelines on the care of patients with an abdominal aortic aneurysm. J Vasc Surg.

[20] Goff DA, Mangino JE, Glassman AH, Goff D, Larsen P, Scheetz R (2020). Review of Guidelines for Dental Antibiotic Prophylaxis for Prevention of Endocarditis and Prosthetic Joint Infections and Need for Dental Stewardship. Clinical Infectious Diseases : Off Pub Infectious Diseases Society Am.

[21] Legout L, Beltrand E, Migaud H, Senneville E (2012). Antibiotic prophylaxis to reduce the risk of joint implant contamination during dental surgery seems unnecessary. Orthopaedics Traumatol, Surgery Res : OTSR.

[22] Moreira AI, Mendes L, Pereira JA (2020). Is there scientific evidence to support antibiotic prophylaxis in patients with periodontal disease as a means to decrease the risk of prosthetic joint infections?. A Systematic Rev Internat Orthopaedics.

[23] Kao FC, Hsu YC, Chen WH, Lin JN, Lo YY, Tu YK (2017). Prosthetic Joint Infection following invasive dental procedures and antibiotic prophylaxis in patients with hip or knee arthroplasty. Infect Control Hosp Epidemiol.

[24] Skaar DD, O'Connor H, Hodges JS, Michalowicz BS (2011). Dental procedures and subsequent prosthetic joint infections: findings from the medicare current beneficiary survey. J Ame Dental Associat.

[25] Thornhill MH, Crum A, Rex S, Stone T, Campbell R, Bradburn M, Fibisan V, Lockhart PB, Springer B, Baddour LM, Nicholl J (2022). Analysis of prosthetic joint infections following invasive dental procedures in England. JAMA Netw Open.

[26] Millot S, Lesclous P, Colombier ML, Radoi L, Messeca C, Ballanger M, Charrier JL, Tramba P, Simon S, Berrebi A, Doguet F, Lansac E, Tribouilloy C, Habib G, Duval X, Iung B (2017). Position paper for the evaluation and management of oral status in patients with valvular disease: Groupe de Travail Valvulopathies de la Société Française de Cardiologie, Société Française de Chirurgie Orale, Société Française de Parodontologie et d'Implantologie Orale, Société Française d'Endodontie et Société de Pathologie Infectieuse de Langue Française. Arch Cardiovasc Dis.

[27] Ziebolz D, Rost C, Schmidt J, Waldmann-Beushausen R, Schöndube FA, Mausberg RF, Danner BC (2018). Periodontal bacterial DNA and their link to human cardiac tissue: findings of a pilot study. Thorac Cardiovasc Surg.

[28] Terezhalmy GT, Safadi TJ, Longworth DL, Muehrcke DD (1997). Oral disease burden in patients undergoing prosthetic heart valve implantation. Ann Thorac Surg.

[29] Šutej I, Peroš K, Trkulja V, Rudež I, Barić D, Alajbeg I, Pintarić H, Stevanović R, Lepur D (2020). The epidemiological and clinical features of odontogenic infective endocarditis. Eur J Clinical Microbiol Infectious Diseases: Off Publicat Europ Society Clinical Microbiol.

[30] Strom BL, Abrutyn E, Berlin JA, Kinman JL, Feldman RS, Stolley PD, Levison ME, Korzeniowski OM, Kaye D (2000). Risk factors for infective endocarditis: oral hygiene and nondental exposures. Circulation.

[31] Zawadzki PJ, Perkowski K, Padzik M, Mierzwińska-Nastalska E, Szaflik JP, Conn DB, Chomicz L (2017). Examination of oral microbiota diversity in adults and older adults as an approach to prevent spread of risk factors for human infections. Biomed Res Int.

[32] Danilkowicz RM, Lachiewicz AM, Lorenzana DJ, Barton KD, Lachiewicz PF (2021). Prosthetic joint infection after dental work: is the correct prophylaxis being prescribed? a systematic review. Arthroplasty today.

[33] Teixeira, E. C., Warren, J. J., McKernan, S. C., McQuistan, M. R., Qian, F. (2020). Prescribing practices for antibiotic prophylaxis in patients with prosthetic joints. Special care in dentistry : official publication of the American Association of Hospital Dentists, the Academy of Dentistry for the Handicapped, and the American Society for Geriatric Dentistry 40 (2) 198–205. 10.1111/scd.12450 10.1111/scd.12450 31965592

[34] Skaar DD, Park T, Swiontkowski MF, Kuntz KM (2019). Is Antibiotic prophylaxis cost-effective for dental patients following total Knee arthroplasty?. JDR Clinical Translational Res.

[35] Ogawa M, Satomi-Kobayashi S, Yoshida N, Tsuboi Y, Komaki K, Nanba N, Izawa KP, Sakai Y, Akashi M, Hirata KI (2021). Relationship between oral health and physical frailty in patients with cardiovascular disease. J Cardiol.

