# Supplementary material for: Exploring the link between poor oral hygiene and mesh infection after hernia repair: a systematic review and proposed best practices
Source: Hernia. 2023 May 19;27(6):1387–95. doi: 10.1007/s10029-023-02795-y (PMC10700451; doi:10.1007/s10029-023-02795-y)
Supplement: Supplementary file 1 — Supplementary file1 (DOCX 17 KB) [file 10029_2023_2795_MOESM1_ESM.docx]

| **Supplementary table 1:** Excluded studies after reading full text / reason for exclusion | | | | |
| --- | --- | --- | --- | --- |
|  |  |  |  |  |
| **Author** | **Year** | **Name of article** | **Type of study** | **Reason for exclusion** |
| Danilkowicz et al. | 2021 | Prosthetic Joint Infection After Dental Work: Is the Correct Prophylaxis Being Prescribed? A Systematic Review | SR | Different outcome |
| Goff et al. | 2020 | Review of Guidelines for Dental Antibiotic Prophylaxis for Prevention of Endocarditis and Prosthetic Joint Infections and Need for Dental Stewardship | review of guidelines | Different outcome |
| Guay et al. | 2012 | Antimicrobial prophylaxis in noncardiac prosthetic device recipients. | SR | Old SR, newer one available |
| Gundre et al. | 2011 | Prosthetic valve endocarditis caused by Gemella sanguinis: a consequence of persistent dental infection. | case report | Wrong study type |
| Kinane et al. | 2005 | Bacteraemia following periodontal procedures. | Prospective cohort study | Different topic |
| Lam et al. | 2013 | Is it safe to perform dental and cardiac valve surgeries concomitantly? | retrospective cohort study | Different topic |
| Lockhart et al. | 2008 | Bacteremia asso-ciated with tooth brushing and dental extraction. | Prospective cohort study | Different topic |
| Lowry et al. | 2005 | Hearts and mouths: perceptions of oral hygiene by at-risk heart surgery patients. | qualitative study | Different topic |
| Mincer et al. | 2021 | Pre-Cardiac Dental Treatment Does Not Increase the Risk of Adverse Events. | retrospective record study | Different topic |
| Moreira et al. | 2020 | Is there scientific evidence to support antibiotic prophylaxis in patients with periodontal disease as a means to decrease the risk of prosthetic joint infections? A systematic review | SR | No outcomes, all studies excluded |
| Noori et al. | 2019 | Is Antibiotic Prophylaxis Necessary Before Dental Procedures in Patients Post Total Ankle Arthroplasty? | SR | Different topic |
| Ogawa et al. | 2021 | Relationship between oral health and physical frailty in patients with cardiovascular disease | retrospective cohort study | Different topic |
| Rakow et al. | 2019 | Origin and characteristics of haematogenous periprosthetic joint infection | retrospective cohort study | Different topic |
| Rao et al. | 2020 | Preoperative dental screening prior to cardiac valve surgery and 90-day postoperative mortality. | retrospective cohort study | Different topic |
| Rieber et al. | 2019 | Slackia exigua, an anaerobic Gram-positive rod and part of human oral microbiota associated with periprosthetic joint infection of the hip. First case and review of the literature. | case report | Wrong study type |
| Ross et al. | 2018 | Outbreak of bacterial endocarditis associated with an oral surgery practice: New Jersey public health surveillance, 2013 to 2014. | case series | Different topic |
| Skaar et al. | 2018 | Is Antibiotic Prophylaxis Cost-effective for Dental Patients Following Total Knee Arthroplasty? | cost-effectiveness analysis | Wrong study type |
| Sonohata et al. | 2014 | Acute Hematogenous Infection of Revision Total Hip Arthroplasty by Oral Bacteria in a Patient without a History of Dental Procedures: Case Report. | case report | Wrong study type |
| Strom et al. | 2000 | Risk factors for infective endocarditis: oral hygiene and nondental exposures. | retrospective cohort study | Different topic |
| Šutej et al. | 2020 | The epidemiological and clinical features of odontogenic infective endocarditis. | retrospective cohort study | Different topic |
| Terezhalmy et al. | 1997 | Oral disease burden in patients undergoing prosthetic heart valve implantation | Prospective cohort study | Very old |
| Yasny et al. | 2009 | Dental considerations for cardiac surgery. | review | Old review |
| Zawadzki et al. | 2019 | Examination of Oral Microbiota Diversity in Adults and Older Adults as an Approach to Prevent Spread of Risk Factors for Human Infections. | retrospective cohort study | Different topic |
| Ziebholz et al. | 2018 | Periodontal Bacterial DNA and Their Link to Human Cardiac Tissue: Findings of a Pilot Study. | Prospective cohort study | Different topic |
|  |  |  |  |  |
