# Supplementary material for: Exploring the link between poor oral hygiene and mesh infection after hernia repair: a systematic review and proposed best practices
Source: Hernia. 2023 May 19;27(6):1387–95. doi: 10.1007/s10029-023-02795-y (PMC10700451; doi:10.1007/s10029-023-02795-y)
Supplement: Supplementary file 2 — Supplementary file2 (DOC 43 KB) [file 10029_2023_2795_MOESM2_ESM.doc]

**Supplementary Table 2:** **Risk of Bias in the Published Studies** (By the Risk Of Bias In Non-Randomised Studies – of Interventions - ROBINS-I Tool).

|  |  | ***Pre-intervention*** | | ***At intervention*** | ***Post-intervention*** | | | | ***Final Judgement*** |
| --- | --- | --- | --- | --- | --- | --- | --- | --- | --- |
| **Authors** | **Study type** | **Bias due to confounding** | **Bias in selection of participants into the study** | **Bias in classification of interventions** | **Bias due to deviations from intended interventions** | **Bias due to missing data** | **Bias in measurement of outcomes** | **Bias in selection of the reported results** |  |
| Konstanty-Kalandyk 2019 | Prospective cohort study | Low | Low | Low | Low | Low | Low | Low | Low |
| Pedersen 2019 | Prospective cohort study | Low | Moderate | Moderate | Moderate | Low | Low | Low | Moderate |
| Suzuki 2019 | Prospective cohort study | Low | Moderate | Low | Low | Low | Low | Low | Moderate |
| Nikishawa 2020 | Prospective cohort study | Low | Low | Low | Low | Low | Low | Low | Low |
| Nobuhara 2018 | Retrospective cohort study | Moderate | High | Low | Low | Moderate | Moderate | Moderate | Moderate |
| Nikiskawa 2019 | Retrospective cohort study | Low | Low | Low | Low | Moderate | Low | Low | Low |
| Hasegawa 2021 | Retrospective cohort study | Low | Moderate | Low | Low | Moderate | Low | Low | Low |
| Nobuhara 2022 | Retrospective cohort study | Low | Low | Low | Low | Moderate | Low | Low | Low |
| Skaar 2011 | Retrospective cohort study | High | High | High | Moderate | High | High | High | High |
| Kao 2017 | Retrospective case-control study | High | High | High | Moderate | Moderate | High | High | High |
| Thornhill 2022 | Retrospective cohort study | Very high | Very high | Very high | Moderate | Very high | High | High | Very high |
