# Supplementary material for: Exploring the link between poor oral hygiene and mesh infection after hernia repair: a systematic review and proposed best practices
Source: Hernia. 2023 May 19;27(6):1387–95. doi: 10.1007/s10029-023-02795-y (PMC10700451; doi:10.1007/s10029-023-02795-y)
Supplement: Supplementary file 3 — Supplementary file3 (DOCX 19 KB) [file 10029_2023_2795_MOESM3_ESM.docx]

| **Supplementary table 3: A list of included systematic reviews and guidelines related to the topic of dental/oral health/hygiene and risk of implant infection.** SR – systematic review, MA – meta-analysis, ATB – antibiotic, IE - infective endocarditis, EVAR - Endovascular Aneurysm Repair, OSR - open surgical repair, CVS - cardiac valve surgery, LVAD - left ventricular assist device. RR - risk ratio, CI - confidence interval  Milot et al. and Chaikof et al. are two Guidelines we have used a statement from one of their chapters. For completeness, we have kept them in this table. | | | | | | | |
| --- | --- | --- | --- | --- | --- | --- | --- |
|  |  |  |  |  |  |  |  |
| **Author** | **Year** | **Name of article** | **Type of study** | **Size** | **Results** | **Main findings** | **Quality of evidence** |
| Millot et al. | 2017 | Position paper for the evaluation and management of oral status in patients with valvular disease: Groupe de Travail Valvulopathies de la Société Française de Cardiologie, Société Française de Chirurgie Orale, Société Française de Parodontologie et d'Implantologie Orale, Société Française d'Endodontie et Société de Pathologie Infectieuse de Langue Française. | guideline |  | Incidence of IE in France 3/100.000 (2000cases/year). Treatment = prolonged i.v. ATB, 50% valvular surgery during acute phase. Mortality: 20% in hospital, 40% at 4 years. Responsible microorganisms - oral streptococci (19%), staphylococci (35%). Low-grade but repeated bacteraemia occurs during daily life activities and may cause IE on diseased valves. These daily events are not amenable to antibiotic prophylaxis, but depend on oral hygiene, and represent a rationale for the detection and treatment of all infectious oral foci in patients at risk of IE. | Periodontal management involves non-surgical therapy (oral health education, scaling, root planning, maintenance). Maintenance of oral hygiene by regular scaling may reduce the risk of infectious diseases. The working group proposes periodontal treatment before surgery. Thorough search for and treatment of oral infectious foci are needed before valvular intervention. |  |
| Chaikof et al. | 2018 | The Society for Vascular Surgery practice guidelines on the care of patients with an abdominal aortic aneurysm. | guideline |  | ATB prophylaxis to prevent graft infection before any dental procedure involving the manipulation of the gingival or periapical region of teeth or perforation of the oral mucosa, including scaling and root canal procedures, for any patient with an aortic prosthesis, whether placed by OSR or EVAR **STRONG recommendation** | Any potential sources of dental sepsis to be eliminated at least 2 weeks before implantation of a prosthetic valve or other intracardiac or intravascular foreign material, unless the procedure was deemed urgent. **STRONG recommendation** |  |
| Barrere et al. | 2019 | Dental assessment prior to orthopedic surgery: A systematic review. | SR | 19057 | 12 case series, 4 case-control studies and 12 cohort studies. 6/224 of cases (2.9%) had dental abscess in the case-control studies. Only 4 cohort studies reported on preoperative examination – scaling –polishing 78/205 (38%), extraction 49/205 (24%), restorative work 37/205 (18%). | Infectious complication less frequent if preoperative examination has been performed. Substantial heterogeneity among included studies. No formal evidence for or against preoperative dental assessment, but it is advisable to perform this with the aim of maintaining favourable oral hygiene and thus reduce the risk factors. | low |
| Lockhart et al. | 2019 | Effect of dental treatment before cardiac valve surgery: Systematic review and meta-analysis. | SR and MA | 1598 | Cardiac valve surgery (CVS) and left ventricular assist device (LVAD) implantation. Six studies met inclusion criteria for CVS but none for LVAD implantation. | There is very low certainty evidence on whether dental treatment before CVS affects postsurgical outcomes, including all-cause mortality (RR, 1.00; 95% CI, 0.53 to 1.91), infective endocarditis (RR, 1.30; 95% CI, 0.51 to 3.35), postsurgical infection (RR, 1.01; 95% CI, 0.76 to 1.33), and hospital stay (weighted mean difference, 2.9; 95% CI, -2.3 to 8.1). Dentists and medical professionals should collaborate to determine the best course of action for each patient, considering potentially relevant care. | low |
| Legout et al. | 2012 | Antibiotic prophylaxis to reduce the risk of joint implant contamination during dental surgery seems unnecessary | SR | Unknown | 68 articles looking into: frequency and intensity of bacteraemia of orodental origin, frequency of implant infection secondary  to dental surgery, and objective efficacy of antibiotic  prophylaxis in dental surgery in patients with joint implants. | risk of onset of bacteraemia is 154,000-fold greater in everyday activities than in tooth extraction, orodental hygiene is important for  the prevention of implant infection, then ATB prophylaxis prior tooth extraction | low |
