# Supplementary material for: Exploring the link between poor oral hygiene and mesh infection after hernia repair: a systematic review and proposed best practices
Source: Hernia. 2023 May 19;27(6):1387–95. doi: 10.1007/s10029-023-02795-y (PMC10700451; doi:10.1007/s10029-023-02795-y)
Supplement: Supplementary file 4 — Supplementary file4 (DOCX 16 KB) [file 10029_2023_2795_MOESM4_ESM.docx]

| **Table 4: A list of included prospective studies related to the topic of dental/oral health/hygiene and risk of implant infection.** CABG - coronary artery bypass graft, MRSA - methicillin resistant Staphylococcus aureus, NNT - number needed to treat, ARR - absolute risk reduced, PD - periodontal disease, IE - infective endocarditis, EVAR - EndoVascular Aneurysm Repair, OSR - open surgical repair, CVS - cardiac valve surgery, LVAD - left ventricular assist device. RR - risk ratio, CI - confidence interval, BMI - body mass index, TT - temperature, DM - diabetes mellitus, OR – odds ratio | | | | | | | | | |
| --- | --- | --- | --- | --- | --- | --- | --- | --- | --- |
| **Author** | **Year** | **Name of article** | **Type of study** | **Size** | **Study design** | **Main findings** | **Risk of bias** | **Quality of evidence** |  |
| Konstanty-Kalandyk et al. | 2016 | Incomplete oral sanation as a risk factor for elevated leucocytosis and postoperative infection. | Prospective cohort study | 240 | A (n=185) patients received dental treatment to eradicate inflammatory foci (Conservative: 70%, Endodontic: 30%, and/or Surgical: 85%). B (n=55) interrupted oral sanation due to symptoms. 55.9% had CABG and 34.7% had valve replacement. Average age 60 years, DM 27.6%, MRSA+ in nose swab 26.7%. Sanation achieved in 70% of CABG and 83% of valve patients. Overall, 59% reported regular dentist visits, 34% didn't go, and 36% brushed teeth once a day or less | More infections in group B (4/55 vs. 3/185; 7.27% vs. 1.62%; p = 0.062); Leucocytosis levels were higher in group B (10.96 vs.10.11 × 103/μL, p = 0.059). After removing all cases of infection in both groups, the level of leucocytosis remains higher in group B (10.98 vs. 10.13 × 103/μL, p = 0.061). Independent risk factors for infection were incomplete oral sanation (OR 6.1, p = 0.042) and diabetes mellitus (OR 5.38, p = 0.059) in the preoperative period. | low | low |  |
| Pedersen et al. | 2019 | Preoperative oral hygiene recommendation before open-heart surgery: patients' adherence and reduction of infections: a quality improvement study. | Prospective cohort study | 972 | CVS, control group (506), intervention group (466). Intervention = teeth and mouth cleaning recommendation. 86.9% (95% CI 83.3 to 89.8) adhered to recommendation, no differences in gender, age, smoking, alcohol, surgery type or BMI. Fewer ATB prescriptions on 5th post-op day in intervention group (p<0.015). RR=0.65 (95% CI 0.48 to 0.96), NNT=22.0, RRR=0.52, ARR=0.042. For adherent patients RR=0.49 (95% CI 0.31 to 0.77), NNT=15.9, RRR=1.01, ARR=0.063. | In the intervention group, antibiotics use on the fifth postoperative day decreased by 34%, and by 50% in patients who adhered to the recommendation. The reduction was seen in all types of infections, but not significant due to low incidence. Deep SSI incidence was lower in the control group 6/1.2% vs intervention group 2/0.4%. | moderate | low |  |
| Suzuki et al. | 2019 | Preoperative periodontal treatment and its effects on postoperative infection in cardiac valve surgery. | Prospective cohort study | 102 | 64 patients underwent preoperative periodontal treatment, 38 historical controls. Days of TT above 37,5C after CVS measured. | The number of days of high fever was significantly lower in the intervention group than in the control group (p= 0.01). | moderate | low |  |
| Nishikawa et al. | 2020 | The bacterial association with oral cavity and intra-abdominal abscess after gastrectomy. | Prospective cohort study | 52 | 52 people undergoing gastric resection. Bacteria in oral cavity and in stomach mucosa compared. It is hypothesized that patients with severe PD would swallow a much larger number of bacteria than those with mild PD. | Bacterial cultures from the stomach mucosa were positive in 26 cases (50%) despite the administration of prophylactic antibacterial drugs before surgery. Bacteria were not detected in the stomach mucosa of patients with mild PD. | low | low |  |
