# Supplementary material for: Exploring the link between poor oral hygiene and mesh infection after hernia repair: a systematic review and proposed best practices
Source: Hernia. 2023 May 19;27(6):1387–95. doi: 10.1007/s10029-023-02795-y (PMC10700451; doi:10.1007/s10029-023-02795-y)
Supplement: Supplementary file 5 — Supplementary file5 (DOCX 18 KB) [file 10029_2023_2795_MOESM5_ESM.docx]

**Supplementary table 5: A list of included prospective studies related to the topic of dental/oral health/hygiene and risk of implant** infection. CABG - coronary artery bypass graft, MRSA - methicilin resistant Staphylococcus aureus, NNT - number needed to treat, ARR - absolute risk reduced, PD - periodontal disease, IE - infective endocarditis, EVAR - EndoVascular Aneurysm Repair, OSR - open surgical repair, CVS - cardiac valve surgery, LVAD - left ventricular assist device. RR - risk ratio, CI - confidence interval, BMI - body mass index, TT - temperature, DM - diabetes mellitus, PJI - prosthetic joint infection, IDP - invasive dental procedure, LPJI - latent prosthetic joint infection, OR - odds ratio, HR - hazard ratio

| **Author** | **Year** | **Name of article** | **Type of study** | **Size** | **Study design** | **Main findings** | **Risk of bias** | **Quality of evidence** |
| --- | --- | --- | --- | --- | --- | --- | --- | --- |
| Nobuhara et al. | 2018 | Effect of perioperative oral management on the prevention of surgical site infection after colorectal cancer surgery | retrospective cohort study | 689 | 563 patients received perioperative oral management (oral management group) and 135 did not (control group) | More SSI in the non-management group. OR of the oral management group 0.484 (P = .014; 95% confidence interval: 0.272–0.862). Mean postoperative hospital stay significantly shorter. | Moderate | Low |
| Nishikawa et al. | 2019 | Clinical impact of periodontal disease on postoperative complications in gastrointestinal cancer patients | retrospective cohort study | 341 | Single institution historical study. Dentists assessed the oral environment for periodontal disease, hygiene status, dry mouth, fur on tongue, and tooth stumps. All patients received scaling and tooth brushing instructions. | A logistic regression analysis identifed periodontal disease as an independent risk factor for POICs, and the OR was 2.091 (p=0.037, 95% confdence interval 1.045–4.183). | Low | Moderate |
| Hasegawa et al. | 2021 | Effects of preoperative dental examination and oral hygiene instruction on surgical site infection after hepatectomy: a retrospective study. | retrospective cohort study | 334 | Retrospective analysis of records of patients undergoing liver resections for cancer. | Risk of SSI: bacterial infection of ascites (OR = 13.72), lack of preoperative oral management intervention (OR = 10.17), and severe liver fibrosis (OR = 2.76). | Low | Moderate |
| Nobuhara et al. | 2022 | Perioperative oral care can prevent surgical site infection after colorectal cancer surgery: A multicenter, retrospective study of 1,926 cases analyzed by propensity score matching | retrospective cohort study | 1480 | 1,926 patients with colorectal cancer from 8 institutions; 808 (oral care group): perioperative oral care at the hospital’s dental clinic, 1,118 (control group) did not receive perioperative oral care. The data were matched by propensity score to reduce bias. 1,480 patients were included in the analysis. | Incidence of SSI significantly lower in the oral care group than in the control group (8.4% vs 15.7%, P < .001). Multivariate logistic regression analysis revealed 4 independent RF for surgical site infection: low albumin level, rectal cancer, blood loss, and lack of perioperative oral care. Lack of perioperative oral care had an OR of 2.100 (95% confidence interval 1.510e2.930, P < .001). | Low | Moderate |
| Skaar et al. | 2011 | Dental procedures and subsequent prosthetic joint infections: findings from the Medicare Current Beneficiary Survey | retrospective cohort study | 168 | 1997-2006 data from the Medicare Current Beneficiary Survey (MCBS), participants who had undergone total joint arthroplasty and those who had experienced a PJI. Associations between dental procedures and subsequent PJIs - time-to-event analyses (N = 1,000). A nested case-control study included case participants who had had PJIs (n = 42) and matched control participants who had had total arthroplasty but had no PJIs (n = 126). | Control participants were more likely to have undergone an invasive dental procedure, though this trend was not statistically significant in either the time-to-event analysis (HR = 0.78; 95 percent confidence interval [CI], 0.18-3.39) or the case-control analysis (OR = 0.56; 95 percent CI, 0.18-1.74). Only four of 42 case participants had undergone an invasive dental procedure in the 90 days before the infection occurred. Consideration of all dental procedures yielded similar results. | High | Very low |
| Kao et al. | 2017 | Prosthetic Joint Infection Following Invasive Dental Procedures and Antibiotic Prophylaxis in Patients With Hip or Knee Arthroplasty | Retrospective case-control study | 13026 | All Taiwaneese resipients after hip and knee arthroplasty were screened. 57,066 had dental workup. They were individually matched 1:1 with the nondental cohort by age, sex, propensity score, and index date. The dental cohort was further divided by the use or nonuse of prophylactic antibiotics. The antibiotic and nonantibiotic subcohorts comprised 6,513 matched pairs. | PJI - 328 patients (0.57%) in the dental subcohort and 348 patients (0.61%) in the nondental subcohort, no between-cohort difference 1-year cumulative incidence (0.6% in both, P=.3). Multivariate-adjusted Cox regression - no association between dental procedures and PJI. PJI occurred in 13 patients (0.2%) in the ATB+ and 12 patients (0.18%) in the ATB- (P=.8). Multivariate-adjusted analyses confirmed that there was no association between the incidence of PJI and prophylactic antibiotics. | High | Very low |
| Thornhill et al. | 2022 | Analysis of Prosthetic Joint Infections Following Invasive Dental Procedures in England | retrospective cohort study | 9427 | Case-crossover and time trend design to examine any potential association between IDP and LPJI. All cases of hospital admission with PJI and known dental records were analysed. Groups of patients who had an invasive dental procedure up to 3 months before the admisssion and those who had no invasive dental procedure were compared. | 9427 LPJI hospital admissions with dental records identified. 2385 (25.3%) had hip prosthetic joints, 3168 (33.6%) had knee prosthetic joints, 259 (2.8%) had other prosthetic joints, and 3615 (38.4%) had unknown prosthetic joint types. There was no significant temporal association between IDP and subsequent LPJI. Indeed, there was a lower incidence of IDP in the 3 months prior to LPJI (incidence rate ratio, 0.89; 95%CI, 0.82-0.96; P = .002). | High | Very low |
|  |  |  |  |  |  |  |  |  |
|  | | | | | | | |  |
